# Digital Life Models and Transformative Choices: Could AI Simulations of Individual Minds (AI SIMs) Help us Make Important Life Decisions More Rationally and Authentically?

**DOI:** 10.1007/s12152-026-09662-4

**Published:** 2026-08-15

**Authors:** Christopher Register, Sebastian Porsdam Mann, Cristina Voinea, Julian Koplin, Julian Savulescu, Brian D. Earp

**Affiliations:** 1 Uehiro Oxford Institute, University of Oxford, Oxford, United Kingdom; 2 Center for Advanced Studies in Bioscience Innovation Law, University of Copenhagen, Copenhagen, Denmark; 3 Monash Bioethics Centre, Monash University, Melbourne, Australia; 4 Centre for Biomedical Ethics, Yong Loo Lin School of Medicine, National University of Singapore, Singapore, Singapore

**Keywords:** AI SIMs, Digital life model, Personal forecasting, Transformative choices

## Abstract

The article advances the idea of "digital life models" to assist with difficult personal decisions, including those that can change an individual in profound ways. We propose that if personalized AI simulations can model how an individual's life might unfold under different conditions, this could help the individual better understand the likelihoods and expected subjective value of these possible outcomes. It remains to be seen whether these digital life models are technically feasible, given fundamental challenges in modeling psychological complexity, person-environment interactions, and value change. Ethical concerns include data privacy, cultural bias, and the risk of improperly shaping user decisions. Acknowledging these limitations, we argue that digital life models, if successfully developed, could in principle enhance rational and authentic decision-making in the face of potentially transformative change.

##  Introduction

Some of the most significant decisions we face are also the hardest to make. Choices such as whether to become a parent, change career, or emigrate can change not only what we know, but who we are. They can also change the values by which we assess our lives. L. A. Paul [1] calls these transformative choices: choices about whether to undergo an experience that is both *epistemically* transformative (one cannot know in advance what it will be like), and *personally* transformative (it may change one's deepest values and commitments). 

Standard rational choice models say it’s rational to make choices that have the highest expected value [1–4]. But this is easier said than done when the options involve deep conflicts between values we care about. Things become even more complicated when the choice itself might change who we are. If becoming a parent or switching careers could change our deepest values, then the person who makes the choice and the person who lives with its consequences may not share the same set of values; so there is no stable point from which to calculate which option is best. 

Some philosophers have argued that we are not entirely without resources: statistics, the testimony of others who have faced similar choices, and imaginative projection of our possible futures can each, in principle, support rational decision-making even in transformative cases [5–8]; see also [9]. But others have pushed back, arguing that statistical information and testimony can only ever get us so far (e.g., [10]; see also [11]): after all, what is true on average or for others, may not be true for *us*, specifically. And imaginative projection too can be limited by ignorance or distorted by our current mood and emotional state, making it an unreliable guide to how we will actually feel once a choice is made [12, 13].

In this paper, we explore an emerging possibility for better informing difficult life decisions. We introduce the notion of a ‘digital life model’ (DLM), which is still a hypothetical, but we think theoretically viable, technology. A DLM would combine a personalized AI simulation of an individual mind, a context-sensitive model of plausible future life-paths, and forecasting tools to simulate how a particular person might respond to a transformative choice. Our central claim is that DLMs, if successfully developed, could in principle help people make transformative decisions that are better informed, more rational, and more authentic than what existing methods allow, while also introducing risks that would need to be carefully managed. What makes DLMs helpful for transformative choices is that they could provide individual-level rather than (solely) population-level forecasting, simulated first-personal perspectives of possible future selves rather than (solely) third-personal descriptions of others' experiences, and explicit modeling of how values might change under each possible outcome. 

Some caveats before we proceed. First, this paper advances an idea, so it is conceptual in nature, rather than technical; more specifically, we do not propose a technical blueprint for DLMs, nor will we show how to overcome all of the design challenges DLMs face. Second, we focus on *transformative* choices because they pose especially intractable epistemic problems for personal decision-making. They are a valuable test case for the potential usefulness of DLMs. We also focus primarily on *paradigmatic* cases of transformative choices such as decisions to become a parent, start a new career, or move to a new country [14]. This is because it is more likely that there will be suitable population-level data about how people tend to respond to these choices that could be combined with individual-level data. We begin with a brief overview of the nature of transformative decision-making, showing why such decisions are so difficult. We then summarize current research suggesting that DLMs *might* be technically feasible, although our focus in this article is, as mentioned, on conceptual arguments. However, there are important design challenges, epistemic limitations, and ethical concerns that will arise if and when DLMs are developed for this purpose. After discussing these concerns, we close by suggesting ways forward.

## Why Transformative Choices Are So Difficult

When we face life-changing decisions, we often seek information so that we can make a well-informed judgment about each available option. A well-informed judgment should be based on information about:

- **Current values**: how, for a given possible outcome, that outcome promotes or demotes what the person currently values;
- **Likelihoods**: the likelihoods of possible outcomes, including likelihoods for how the personʼs values themselves may change;
- **Future values**: how a person would evaluate an outcome from the standpoint of their future selves.^1^

Three standard sources for acquiring this information are statistics from social science, personal testimony of others who have undergone similar experiences, and imaginative projections from one’s past experiences. Each faces limitations in the context of transformative choices [1, 10], limitations that, we will argue, a well-designed DLM could at least partially address.

### The Problem of Applicability

Social-scientific data describe population-level trends. Knowing the average life satisfaction of new parents offers limited guidance about how a *specific individual*, with their unique psychology, history, and circumstances, will fare. As Paul [10] notes, population-level statistics may be accurate for the group while remaining poor estimates for any given member of it;^2^ this is precisely because statistics abstract away from the individual variance that could make a difference. Testimony from friends or family often faces the same problem (but see [15] on intimate, second-personal testimony from close others). It comes from individuals whose first-personal experiences, values, and so on, may not mirror one's own. Relying on such evidence can be misleading: just because one's peers report joy in a particular career choice, becoming a parent for the first time, or whatever, does not guarantee a similar outcome for oneself. Finally, and perhaps more surprisingly, information provided by imaginative projection may also be a poor candidate for predictions about how one would in fact respond to an outcome. When assessing outcomes by one’s current values, imaginative projection is sometimes influenced by extraneous factors such as one’s current mood or emotional state, which distort affective forecasting [12]. So, the predictions one draws from imagining a future outcome can be a poor guide to how one will actually evaluate that outcome once it arrives.

It seems that the three sources of information cannot reliably deliver information of types (a), (b), or (c) as applied to *a specific* person in *specific* circumstances.

### The Problem of Third-personal Format

Even if the specific applicability problem could be solved (say, by finding statistics derived from people very similar to oneself), a further issue remains. Statistics and testimony deliver objective descriptions of others' reactions: what people report, how they behave, what they say they value. What they cannot provide is first-personal insight into what an outcome will be *like for oneself*, the phenomenological perspective that Paul [1] argues is central to authentic decision-making. To decide authentically, on her view, one must act on reasons whose significance one can grasp from one's own evaluative standpoint, rather than by extrapolating from what relevantly similar others have experienced. This gap between third-personal information and first-personal knowledge persists even when the third-personal data is specifically applicable to the individual. The gap tracks a difference between two standards a decision might be held to. A decision may be rational, that is, maximizing expected utility, without being authentic in Paul's sense [16]. It is authenticity, not rationality alone, that motivates the search for a more first-personal source of information about transformative outcomes.

### The Problem of Self-alienation

A third limitation concerns value change over time. Transformative choices may change the values by which outcomes are assessed. Take the “Russian Nobleman” thought experiment introduced by Derek Parfit [17] (p. 327): a young Russian nobleman with socialist ideals wishes to distribute his wealth, but anticipates that his values will become more conservative as he ages. It shows how changes of values over time can make our future selves, including their constitutive values, radically different from our present selves (for a different example, involving dementia, see [18]; see also [19].^3^ Such changes pose a problem for choices that may bring them about. Even if one could accurately predict a future self's intense life-satisfaction within a radically different value system, this knowledge might not justify one's current self choosing to transform one's values accordingly. One might know in advance, for example, that joining a cult would result in enthusiastic endorsement of cult life and behavior according to one's future cultish values. But if those values are at odds with one's current values, it may be both irrational and inauthentic, from the perspective of one's present self, to proceed. According to Paul, such a decision risks alienating the ‘ex ante’ self from the ‘ex post’ self [20]; see also [10] (pp. 796-801). Self-alienation of this kind undermines the value, for the current self, of choosing to be changed in the predicted way: why should my current self strive for a future that is valuable only for a future self whose values I do not presently share or endorse?

These three limitations—lack of specific applicability, third-personal format, and the risk of self-alienation—mean that the standard sources of information for transformative choices are, at best, incomplete. What is needed is a source that is individualized rather than population-level, that conveys something of the first-personal character of future outcomes, and that helps the decision-maker assess whether anticipated value changes are ones they can currently endorse. We now turn to whether digital life models could, in principle, provide this.

## Digital Life Models

Artificial Intelligence (AI) has been proposed as a means of improving human decision-making in various spheres, including prudential and moral decision-making based on an individual’s values and circumstances [21–23]. DLMs are another proposal in this tradition. However, they are specifically conceived of as a way of approaching transformative choices, which have been less commonly discussed in the AI literature.

The core idea of a DLM is to use AI and other computational architecture to create a predictive system that combines at least three key features. The first is a personalized AI model of an individual's psychology, which we have elsewhere called an AI SIM (AI Simulation of an Individual Mind) [24]. This is a model trained on individual-level data that aims to capture the individual’s unique constellation of attitudes, beliefs, values, preferences, and personality (e.g., along the lines of [25]). The second is a context-sensitive world model that situates that AI SIM within a structured representation of the circumstances and life paths relevant to the individual's decision (inspired by [26–28]; however, see [29] for critical discussion). The third is a forecasting mechanism that simulates how the individual, as represented by their AI SIM, might respond to a transformative choice across a range of possible outcomes, as represented by the world model. We describe each in turn. 

### The AI SIM

The ability to simulate aspects of people's minds using generative AI and related technologies has been explored in several contexts. Park et al. [25] demonstrated that LLM-based simulations based on interview recordings could replicate participants' survey responses about their attitudes and behaviors with 85% accuracy, which is comparable to the consistency with which humans reproduce their own answers after two weeks. Similarly, Cho et al. [30] reported that LLMs trained on 60 conversational data points achieved 80% accuracy in predicting personal survey responses, with performance improving with more context and training. Starke and colleagues [31] recently published a proof-of-principle study showing that machine learning methods applied to common personal data could predict an individual’s hypothetical treatment preferences more accurately than most human surrogates. And Wright et al. [32] showed that commercially available LLMs can score Big Five personality traits from brief open-ended narratives (streams of thought or daily video diaries), achieving convergence with self-reports at levels comparable to established benchmarks such as self-other agreement and ecological momentary assessment. These findings are significant because they rely on the kind of naturalistic, everyday data that would be more readily available as inputs for an AI SIM than data derived from more complicated or hard-to-access sources, such as specially designed value-eliciting experiments (though these could also be used; see [33] for caveats).

Of course, persons cannot be reduced to such data. However, as Giubilini et al. [22] argue, it is theoretically possible to infer important aspects of a person’s values and preferences from records of how they have behaved and decided in the past. The empirical studies discussed above support this view: just as fine-tuned LLMs have been able to approximate psychological and behavioral dispositions from relatively limited data [25, 30], they could also, potentially, identify patterns that reflect a person’s values and preferences. While these inferences would (like all LLM-based inferences) be limited and probabilistic, they *could* provide a useful model of a person’s normative commitments, particularly when combined with other rich data sources.^4^

### The World Model

After an AI SIM is created, it would need to be exposed to a world model that draws on, and is appropriately structured by, at least two key sources of information. The first source would need to include *general* information about social and institutional environments and how they work, how people tend to behave under various conditions, how values tend to change in response to certain types of experiences, how individual differences bear on these factors, and the nature of multi-agent and person-environment interactions. Broadly speaking, this sort of information would come from existing or future psychosocial and sociological data and associated computer models (see broadly the work of [26–28] with a critical discussion of the limitations of this sort of modeling by, e.g., [29]; see also [34]). A particularly relevant development is recent work showing that LLM agents imbued with distinct value profiles develop trust and relational dynamics in ways that track value similarity between agents. This suggests that world models can, in principle, represent value-relevant social dynamics rather than merely behavioral ones [35].

The second source of information would need to reflect the *particular* circumstances faced by the individual who is considering a transformative decision. For example, someone contemplating parenthood might share their living situation, relationship dynamics, resources and services that are available in their community, realistic career options, and so on. The key is capturing the distinctive context of the transformative choice in question rather than generic scenarios.

### The Forecasting Model

The final step of the process would be to run simulations of how the individual’s life might evolve under different possible outcomes and how their knowledge, preferences, and values would most likely be affected (see, for example, [36] for a preliminary computational model of how a simplified agent would be affected by an epistemic transformation). These simulations would model dynamic interactions between the AI SIM and the world model, such that the AI SIM is modified in response to simulated events and the world model is modified in response to AI SIM behavior. On our proposal, these dynamics would be jointly determined by general and individual information encoded in the AI SIM and the world model. Perhaps this could be done using a Monte Carlo-style simulation engine to model a range of plausible ways a given choice might unfold (for a simplified example using LLM-based agents, see [37]). 

In any case, it would be necessary to systematically vary relevant factors, potentially creating hundreds of simulated “life paths” in a DLM. These simulated biographies would ideally yield a probability distribution over potential futures, analogous to the way this is done in dynamic microsimulation platforms like LifePaths [38]. These computational models simulate the lives of synthetic individuals within virtual populations. By tracking events such as education, employment, marriage, and health changes across thousands of simulated individuals simultaneously, these platforms help policymakers forecast population-level outcomes and evaluate potential interventions [39, 40]; see also [41]. The simulations we propose, however, would also need to reflect *individual* trajectories based on the personalized models that power them.

The outputs of a DLM need not and should not take the form of precise or deterministic predictions. What they can offer is a set of structured possible futures, each described in enough detail to support the kind of deliberation that transformative choices require: not “this is what will happen” but “here is what this kind of future might involve, how it connects to who you are now, and how it would likely be assessed by who you might become.” These conditional, probabilistic predictions can be more or less accurate, even if an individual's eventual choices and life path are not determined in advance. The same holds for forecasting generally: a weather forecast of a 70% chance of rain can be accurate whether or not it rains, since its accuracy depends on how well the stated probability reflects the conditions at the time, and is assessed over many such forecasts, rather than on any single outcome. In the case of a DLM, then, the outputs would be assessable for accuracy in just the same way as any other probabilistic forecast; what would be distinctive is their personalisation. Because personal information is used at each step of the modeling process, and outcomes are assessed by simulated personal values, DLM-based forecasting differs from the population-level statistics and third-personal testimony that currently make up the standard toolkit for transformative choices.

## Digital Life Models as Forecasting Tools

We argue that DLMs, if developed along the lines described in the preceding section, could potentially deliver information of the three types identified above more effectively than existing methods, and so help people make more rational and more authentic choices, including transformative ones. We elaborate on this claim before turning to risks and limitations.

### The Applicability Problem

Recall that the core difficulty with statistics and testimony is that neither delivers information calibrated to you specifically. The most straightforward advantage of DLMs over existing sources therefore concerns specific applicability. Because a DLM draws on data about the particular individual (their psychology, values, circumstances, and the specific choice they face), its forecasts are calibrated to that person rather than derived solely from population averages. Where social-scientific data tell you how the average person fares after becoming a parent or changing career, a DLM would attempt to tell you how you, with your distinctive history, commitments, and context, might fare. This addresses the applicability limitation directly, providing likelihoods—information type (b)—that reflect individual variation (see [42]). The same personalization supports information of type (a): by describing possible outcomes in rich, individualized detail, a DLM can help a user assess which futures resonate with who they currently are, supporting the values-grounded evaluation that authentic deliberation requires.

### The Third-personal Format Problem

Statistics and testimony deliver quantitative or qualitative descriptions of others' experiences. What they cannot deliver is first-personal insight into what an outcome will be like *for you*. A successfully developed DLM could partially bridge this gap. By simulating how the individual's values might evolve under each possible outcome, it can generate outputs written as though from the perspective of each possible future self. The user does not directly subjectively experience these outcomes, but they receive something closer to first-personal testimony from their possible future selves than any existing source provides. This is not perfectly authentic in Paul's sense, because it is a simulation, but decisions informed by it may be *more* authentic than those made through unaided imaginative projection or purely on the basis of third-personal evidence.

### The Self-alienation Problem

How might the risk of self-alienation be addressed through the use of DLMs? The fundamental challenge stems from the fact that not all value shifts are equal. In at least some cases, when one's future values differ from one's current values, it seems irrational and inauthentic to undergo the shift. When I consider joining a cult, to return to that example, I may rationally believe that I am most likely to be happy as a dedicated cult member, based on the testimony of existing cult members. Even so, I know in advance that the sort of values that cult members tend to hold are incompatible with my current values, and so I already have good reason not to proceed. By contrast, if I am contemplating a carefully controlled psychedelic experience statistically associated with increases in well-being and life meaningfulness [43, 44], and I am currently suffering from treatment-resistant depression, say, the risk of self-alienation may be outweighed by concomitant benefits [45]. The key question in both cases is whether the anticipated future values are ones my current self can recognize as a legitimate development of who I already am, rather than a rupture from it. 

Plausibly, DLMs provide resources that could help users determine which side of this line their choice falls on. By generating vivid, personalized descriptions from the perspective of possible future selves, DLMs could support a form of imaginative engagement with future perspectives that might be richer than what statistics or third-party testimony alone could provide (see the use-case in Box 1). This matters because the endorsement question requires something like empathy with one's possible future self. Research on psychological connectedness suggests that such imaginative engagement can itself reduce the felt distance between present and future identity, making endorsement more accessible [46, 47]. The risk of self-alienation cannot be eliminated, but a DLM gives the current self better resources for deciding whether it is a risk worth taking.

Box 1. An illustration of how DLMs could inform deliberation: LLMs, narrative description, and imagination scaffolds

**Table:** 

We suggest that DLMs, as envisioned, would be especially well-positioned to bridge the epistemic-cum-authenticity gap left by purely third-personal data. Because modern AI models, particularly LLMs, excel at generating fluent and richly descriptive text, a DLM could potentially convey information about the *qualities* of a simulated future outcome in accessible, evocative ways [48, 49]. A DLM could tell a story, for example, illustrating how a particular outcome might feel [50], using metaphor and other poetic devices to help the individual imagine what it might be like for them [51]. This is similar to recent proposals to use virtual reality to simulate what it might be like for an individual to undergo a psychedelic experience, specifically so as to enhance the informed consent process (namely, by *scaffolding* the individual’s capacity to imagine what the experience might be like for them in particular, thereby going beyond what they can imagine based solely on others’ testimony) (e.g., [52] see also [53, 54]). Such story-telling could be personalized by drawing analogies to combinations of the user's own past experiences, enabling them to form an imaginative projection. While not inducing the experience itself, this approach could provide richer cognitive and affective anchors than abstract statistics or testimony based on others’ experiences.	

As mentioned, engagement with a DLM need not and should not involve receiving a single predicted trajectory. What it would offer instead is a probability distribution over a structured and conditional set of possible futures: a small number of clustered life paths, each accompanied by a narrative written as though from the perspective of the future self who inhabits it, describing what that life involves, how it feels from the inside, and how that self assesses its situation in light of the values it has come to hold. Where appropriate, these narratives would be accompanied by likelihood ranges rather than precise predictions, and by an explicit account of how each outcome connects to the user's current values and priorities.

A natural worry, however, is that offering a range of simulated possibilities would simply multiply hypotheticals the user cannot meaningfully assess, potentially increasing not insight but decisional paralysis [55, 56]. That could happen if a DLM were poorly designed. A well-designed system should reduce deliberative complexity by, for example, clustering similar outcomes, foregrounding the value tensions most salient for this particular person, and making explicit the assumptions that unaided deliberation tends to leave implicit [57]. Rather than feeling overwhelmed, the user should hopefully end up with fewer and better-organized distinctions to weigh, where the ones that remain would be those that matter most for their decision.

## Technical, Epistemic, and Ethical Concerns

We have argued that, in principle, DLMs could support rational and authentic decision-making in transformative choices better than currently available sources of information. In practice, however, there would be significant technical, epistemic, and ethical challenges. Regarding technical challenges, whether they can be overcome will become clear only if and when substantial engineering and data-scientific effort is devoted to solving them. Regarding epistemic limitations, we’ll argue that these are likely to be more acute in some cases rather than others and that this should affect the manner in which DLMs are used and the extent to which their outputs are relied upon. Regarding ethical concerns, these are most severe if DLMs are adopted uncritically and if they are developed and managed by large institutions or commercial actors. We consider each issue in turn.

### Technical Challenges

Our analysis assumes that DLMs, if developed along the lines described, *could* provide useful forecasts about outcome likelihoods and associated (changes in) values. That assumption may not hold [58]. If these models cannot learn sophisticated patterns of human behavior, or predict psychological change from scientific theories, sociological data, and personal information, they will not generate reliable personalized forecasts. AI capabilities have advanced rapidly, but the complexity of human values, and how they evolve in response to novel experiences, presents serious modeling challenges. Moreover, even rich descriptive outputs may not capture the subjective qualities of experience, which limits their ability to bridge the first-personal knowledge gap central to transformative choices.

Integrating diverse data types, from biographical information to psychological assessments, social network structures, and longitudinal behavioral patterns, also raises hard data-engineering problems (see generally [29]; see also [34]). Mapping between objective life events and subjective experiential qualities is difficult, as is modeling the interactions across life domains that can be central to transformative experiences. The computational resources needed to run thousands of Monte Carlo simulations at sufficient psychological and situational depth would be substantial, raising not only technical and resource challenges but also environmental concerns.

We have pointed to some evidence that DLMs may be technically feasible, but solving these challenges lies beyond our conceptual stage-setting. Substantial engineering and scientific effort would be required before we could know whether the challenges can be met.

### Epistemic Limitations

There are reasons to doubt whether DLMs can ever achieve high predictive accuracy for the proposed purposes. Human lives show sensitive dependence on initial conditions, a form of “chaos” that limits prediction about complex systems [59]. Probabilistic methods that do not purport to track deterministic processes address this partially, but cannot eliminate it. There are also practical limits to the personal information any model can incorporate. Would such systems detect latent neurological factors influencing conditions like adult-onset schizophrenia? Could they predict transformations following profound spiritual experiences? Many life-altering events depend on factors that would be extremely difficult, if not impossible, to model, whatever the algorithmic sophistication.

There is also an *existential* element to choices, related to but distinct from chaotic processes: the expression of freedom of the will. Free will (for those who accept its existence under some plausible description) and subtleties of human values raise two distinct complications. First, it introduces the possibility of forms of self-creation that cannot be wholly determined by, and hence predicted from, antecedent factors. By exercising their will, a person can change how they respond to life-events and how their values evolve.^5^ Second, a person's response may also be shaped by the prediction itself. Once a model presents a range of possible life paths, we may conform to them or rebel against them, sometimes deciding differently just because the predictions were made and we want to assert our freedom. DLMs thus face principled limits in estimating outcome likelihoods in ways that account for free will, agency, and other dynamics of human lives [60, 61].

Relationship or partner-selection decisions are a clear example: outcomes are jointly produced by at least two agents with independent values and degrees of freedom ([15]), and a model’s forecast may alter how a person interprets the other’s signals, manages conflict, or commits to a relationship. For that reason, compared with paradigmatic DLM targets such as career transitions or relocation, where outcomes are more strongly shaped by the user’s own choices within relatively stable structural constraints and better-mapped background regularities, relationship contexts offer a weaker basis for treating model outputs as probability-informed guidance about what could happen. In this context, DLM outputs might be better interpreted as prompts for identifying conditional stressors and value tradeoffs than as grounds for screening in or out a particular relationship. 

Even so, one might worry that forecasting the consequences of a major life decision would require simulating all relevant individuals in a user’s life, including their detailed reactions over time to that decision. If such high-fidelity social modeling were required, large-scale deployment would indeed be infeasible, but DLMs need not require this high level of granularity. Consider the fact that ordinary human deliberation does not typically rely on exhaustively modeling others’ psychologies, but instead is based on coarse-grained expectations, social roles, and observed regularities [62–65]. Likewise, a DLM wouldn’t try to simulate every person in a user’s life in detail. Instead, it would focus on the few people and social factors that actually matter most for the decision, treat the rest of the social world in a simplified way (for example, as typical patterns or rules), and openly represent uncertainty and randomness.

### Interim reflection

Both the technical and epistemic challenges we have identified are substantial, and others may yet be raised by critics and interlocutors. But whether DLMs would be useful does not depend on capturing every causally relevant factor or generating highly accurate forecasts. Ordinary deliberation about transformative choices already proceeds under deep uncertainty, in no small part because of the complexities of human agency. DLMs would not overcome these limits entirely. But they could, in some cases, provide structured insight about conditional risks, plausible trajectories, or value tensions not otherwise obvious or available. This might, for example, include knowledge about how the self could realistically evolve given the constraints and opportunities of one’s environment. Such predictive insight does not require either a deterministic self or a fixed environment. Rather, it assumes that we have selves that are reasonably stable over the near term, and that evolve over longer periods through endogenous and exogenous factors that can be modelled probabilistically (cf. [22]). Given that existing—and quite rudimentary—personalized AI models have shown some success in predicting individual traits and behaviors, better models trained on richer data may allow prediction of more complex and dynamic aspects of human lives. Even then, however, improved prediction would neither replace nor ignore agency. It could help a person better grasp their likely options and associated consequences, without requiring that they defer to such predictions (cf. [61]). Used well in the context of practical deliberation, such information can *support* agency by enabling more knowledgeable self-creation [66].

### Ethical Concerns

Epistemic limitations become ethically significant once we consider how DLMs might be adopted. Without rigorous validation standards, these systems might become popular “life gurus” before demonstrating reliable track records. Some of their proposed advantages, such as narrative fluency and sophistication, could make misleading or biased information more persuasive. And just as recommender systems shape preferences and behavior in ways that serve commercial aims [67], DLMs might similarly nudge life choices toward particular paths that do not serve the user’s own interests. 

Users may also be overly impressed by detailed (if probabilistic) predictions of DLMs, treating those predictions as mandates to be followed rather than as possibilities to be subjected to their own agency. They may then underestimate their ability to shape how they respond to life-events, leading to a fatalist sensibility. A sense of disempowerment could discourage deliberation or the interrogation of DLM outputs.

A related risk is the reinforcement of cultural homogeneity and hegemony. The datasets used to train AI models tend to reflect dominant societal norms and life scripts, such as prioritizing career advancement, financial security, and conventional family structures [68–70]. Even with benign design intentions, a model might favor familiar trajectories and nudge users toward conformity. That could narrow the diversity of life choices and disadvantage those pursuing alternative paths, threatening both individual autonomy and value pluralism. Addressing this risk calls for more than technical fixes: it requires careful thought about how to represent diverse conceptions of flourishing across cultural contexts.

Privacy and data ownership raise further pressing concerns [71–73]. Creating effective digital life models would require unprecedented access to sensitive personal information across cognitive, emotional, and behavioral domains. People increasingly share personal data with digital services (cf. [74]), but the depth and breadth of disclosure needed for accurate life modeling raises serious questions about privacy, security, and informed consent. There are environmental concerns too, as we mentioned: the energy and resources needed to run such models, even if they can be built, would be a heavy cost, one that would need to be justified by gains not achievable through less resource-intensive means. Whether DLMs could meet that standard is far from clear (for a related discussion, see [75]).

These ethical concerns are described in broad terms, and their practical significance will depend on the context in which a digital life model is designed, implemented, and used. Issues of privacy, data ownership, consent, and environmental cost will vary with who controls the model's development and deployment, and how those roles are distributed. At one end of the spectrum, an individual might build and use a DLM privately for their own reflection, curating their own data and relying on existing computational resources, with comparatively limited privacy or environmental implications.

At the other, DLMs could be developed and deployed at scale by powerful institutions or commercial actors, involving extensive data aggregation, centralized control, and heavy computational demands. In between these poles might be commercially developed, open-source general DLMs that are then fine-tuned on personal data, privately and locally, which would at least partly mitigate concerns around privacy and control.

The concept developed here is agnostic about these choices: nothing in our proposal presupposes a particular institutional setting or distribution of control. In practice, however, the default trajectory is one that should raise concerns. Absent organized efforts to support open-source development, local fine-tuning, and strong privacy protections (see, e.g., [76] for a framework), DLMs are likeliest to be built and deployed by large commercial actors. This is also the pathway that, we expect, would carry the most serious ethical risks. Part of our reason for examining DLMs now is that they extend trajectories already visible in current technology. The aim, then, is to assess in advance whether such systems could serve justified decision-making purposes at all—which has been our focus here—before they arrive in whatever form market pressures happen to produce. Ideally, our paper will prompt anticipatory ethical discussion by a wide range of stakeholders, toward identifying which mitigation measures (e.g., data governance requirements, support for decentralized alternatives) could be put in place ahead of commercial deployment, rather than reactively assembled afterward. 

## Ways Forward

The need to get appropriate regulation into place before the development of DLM-like systems is time-sensitive. Recent survey data suggest that seeking personal advice is already a primary use of LLMs in 2025, with millions turning to these systems for guidance on consequential life decisions [77]. Advice-taking from chatbots has also been linked to serious harms [78]. Already in these cases, there is an urgent need for tools designed to support deliberation without nudging users toward undue deference (see, for example, [79] and [66]; cf. [61]). Since DLM outputs could be treated as implicit recommendations, they would need guardrails that keep the system in an exploratory mode: for example, emphasizing uncertainty, presenting trade-offs without arbitrating how they should be handled, and prompting independent user reflection. To reduce the risk of fatalism and disempowerment, outputs should note how actual life paths, compared to merely predicted ones, depend on the user's own agency, and flag decision points as opportunities for self-creation [66]. Described pathways could be framed explicitly as models of options the user may, or may not, choose to enact or pursue.^6^

Importantly, research into DLMs need not proceed in an all-or-nothing fashion. Consider first a DLM built by or for a single individual using their own data and existing computational resources. The achievable level of sophistication here is limited, but so are the ethical risks. Such a system could not generate the richly personalized probabilistic forecasts envisioned in the full proposal, but it could still support structured reflection. Possibly, this could still be a small improvement over unaided deliberation. At the other end of the spectrum, DLMs developed and deployed at scale by organizations or commercial actors open up substantially greater technical possibilities, but also substantially greater ethical stakes. We suggest that early applications should focus on domains where data are most tractable and risks most manageable. Career transitions and educational decisions are natural starting points [80, 81], before expanding to more complex choices as capabilities and governance frameworks ideally develop together.

## Conclusions

In this paper we addressed a mainly conceptual question: if digital life models (DLMs) were successfully developed, could they help individuals bridge the epistemic and evaluative gaps that make big life decisions so difficult? We suggested that, at least in principle, DLMs could help people make choices that are more rational and more authentic, reflecting both their current values and a “scaffolded” grasp of their possible futures. Our aim was not to offer a technical blueprint, but we pointed to existing research suggesting that such models could plausibly be built, in some form, before long. We encourage technical research into designing and testing prototypes, alongside continuing philosophical and ethical work on their appropriate uses, risks, and benefits.

## Data Availability

No datasets were generated or analysed during the current study.

## Footnotes

1 Pettigrew ([82] and [8]) extends theories of rational choice to cases where the agent’s values change over time. Pettigrew’s discussion (especially [8], Ch. 10) suggests these three types of information are important for rational choice. Note also that certainty is not required for any of these types of information in order for a choice to be rational. Suitably constrained uncertainties suffice.

2 Moss ([83], Ch. 9) provides a diagnosis for why statistical evidence is sometimes insufficient for justifying action: the statistical evidence does not constitute knowledge, in particular when one is unable to assign unique probabilities to one’s own future outcomes. This can occur when one is unsure how population-level statistics relate to oneself (for a recent discussion, see [84]; see also [85].

3 We do not here endorse the further claim that deep changes in one’s “self” result in the end of one person’s existence and the beginning of another's, which is of central focus in the philosophy of personal identity. Significant qualitative changes in one’s perspective and values may be compatible with the continuation of personal identity in a numerical sense. See [86]; see also [87] and [88] for discussion.

4 Several technical approaches to creating personalized AI models, or AI SIMs, exist. The prompt-based method used by Park et al. ([25]; see also [89–91]) enhances non-personalized LLMs by providing them with contextual information about an individual through the input prompt, allowing the model to generate personalized outputs without modifying the base model’s internal weights. This approach is efficient and technically straightforward but limited by both prompt length constraints and the persistence of default value embeddings in the base model, which might compete with the information contained in the input prompt. Fine-tuning methods involve secondary training of LLMs on individual-specific data, creating models that attempt to internalize a person's reasoning patterns and values directly into their parameters [30, 92, 93]. Or, as a nearby alternative to LLMs, models could be trained from scratch specifically to model values and preferences, for example by using data on human behavior or psychological change over time rather than linguistic data. Analogously, Savcisens et al. [94] trained a model on data representing sequences of life-events to predict mortality, outperforming traditional methods by 17%—a method that may prove fruitful in modeling other aspects of human life trajectories too. With each strategy, personalized models could be constructed using a person’s explicitly declared values and/or values inferred from past behavior. While such models will inevitably fall short of capturing the full depth of a person’s character or commitments, they may offer workable representations of some aspects of a person’s values.

5 We thank an anonymous reviewer for pressing us on this issue.

6 We thank an anonymous reviewer for this suggestion.
